# Impact of P2Y12 inhibitors on cardiovascular outcomes of Korean acute myocardial infarction patients with baseline thrombocytopenia

**DOI:** 10.3389/fcvm.2022.921955

**Published:** 2022-09-14

**Authors:** Seok Oh, Myung Ho Jeong, Kyung Hoon Cho, Min Chul Kim, Doo Sun Sim, Young Joon Hong, Ju Han Kim, Youngkeun Ahn

**Affiliations:** ^1^Department of Cardiology, Chonnam National University Hospital, Gwangju, South Korea; ^2^Department of Cardiology, Chonnam National University Medical School, Hwasun, South Korea

**Keywords:** antiplatelet drugs, myocardial infarction, percutaneous coronary intervention, Republic of Korea, thrombocytopenia

## Abstract

**Background:**

Antiplatelet therapy is crucial for managing acute myocardial infarction (AMI) and reducing adverse ischemic events after percutaneous coronary intervention (PCI) with drug-eluting stents. However, the ideal P2Y12 inhibitor for patients—particularly East Asians—with AMI and low platelet levels remains unknown. We evaluated the impact of various potencies of P2Y12 receptors on major cardiovascular outcomes of AMI patients with thrombocytopenia in Korea.

**Methods:**

We analyzed the clinical and outcome data of 800 AMI patients with baseline platelet counts <150 × 10^3^/μL who underwent PCI between November 2011 and June 2015. All patient data were obtained from the Korea Acute Myocardial Infarction Registry–National Institutes of Health registry. Subjects were allocated to group A (*n* = 244; treated with potent P2Y12 inhibitors) or group B (*n* = 556; treated with clopidogrel). The primary endpoint was major adverse cardiac and cerebrovascular events (MACCEs).

**Results:**

At the 3-year follow-up, clinical outcomes appeared better in group A than in Group B. However, after propensity score weighting-adjusted analysis, these findings were statistically attenuated, showing a similar incidence of MACCEs between the two groups.

**Conclusions:**

Clopidogrel may be reasonable for patients with low platelet counts and is associated with comparable outcomes to potent P2Y12 inhibitors for Korean AMI patients.

## Introduction

Coronary artery disease (CAD), including acute myocardial infarction (AMI), is recognized as one of the major causes of morbidity and mortality worldwide (1). Many large-scale clinical trials have reported the effectiveness of percutaneous coronary intervention (PCI) for reducing all-cause mortality of AMI patients (2). PCI has become essential in the management of AMI. However, pharmacological treatment after PCI is essential for decreasing the incidence of adverse cardiac events, including stent restenosis and stent thrombosis after PCI (3–8). Dual antiplatelet therapy (DAPT) is a mainstay pharmacological treatment that has evolved and has been refined over the years through many clinical studies. DAPT comprises aspirin—the primary medication—and a P2Y12 inhibitor. The use of P2Y12 inhibitors in this population has significantly reduced the incidence of adverse cardiovascular events (5, 6).

Nevertheless, because ~3.3% of AMI patients are diagnosed with coexisting baseline thrombocytopenia (9), bleeding is a significant concern with DAPT use in these patients. Because specific recommendations regarding the safety of antithrombotic therapy for patients with thrombocytopenia have been hindered by limited evidence, antithrombotic medications are frequently selected based on the clinician's preference and clinical experience. Currently, clinical guidelines offer no clear recommendations regarding antithrombotic therapy for these patients (5, 10).

An increasing body of clinical evidence has shown that East Asians are at lower risk for thrombotic events and higher risk for bleeding complications than Caucasians (11–13). This so-called East Asian paradox has led to the recommendation of different regimens, including reduced doses of antithrombotic agents, for East Asians with cardiovascular disorders (14). This can make it difficult to select optimal antithrombotic agents for East Asian AMI patients with coexisting baseline thrombocytopenia. Therefore, we evaluated the relationship between P2Y12 inhibitor potency and clinical outcomes among AMI patients with baseline thrombocytopenia in the Republic of Korea.

## Materials and methods

### Study population

Participants' data were extracted from the Korean Acute Myocardial Infarction Registry–National Institutes of Health (KAMIR-NIH) from November 2011 to December 2015. The KAMIR-NIH registry is an open, web-based, nationwide, multicenter registry that collects data from 20 tertiary PCI-equipped centers in the Republic of Korea. This nationwide registry contains clinical data regarding the characteristics and outcomes of Korean AMI patients and reflects real-world prognostic and surveillance data.

Among the 13,104 AMI patients in the KAMIR-NIH registry, we identified 800 who equally had thrombocytopenia at baseline. We excluded patients with insufficient data regarding baseline platelet levels, platelet levels ≥150 × 10^3^/μL, who did not undergo PCI, and who did not receive any P2Y12 inhibitors, such as clopidogrel, ticagrelor, or prasugrel. Patients were assigned to one of the two groups based on the type of P2Y12 inhibitor administered. Group A was treated with potent P2Y12 inhibitors (ticagrelor and prasugrel), and group B was treated with clopidogrel. All patients underwent follow-up through regular outpatient visits.

**Figure 1:**
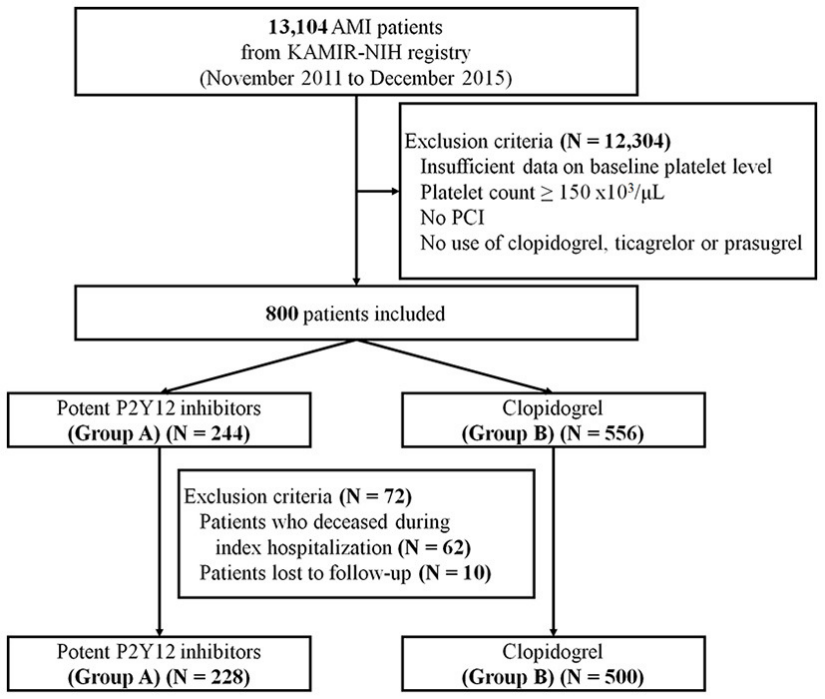
Study scheme. Data of a total of 800 AMI patients from the KAMIR-NIH registry were analyzed. AMI, acute myocardial infarction; KAMIR-NIH, korea acute myocardial infarction registry-national institutes of health; PCI, percutaneous coronary intervention.

This study was conducted according to the ethical principles of the Declaration of Helsinki (15). The study protocol for this registry was previously introduced to and ratified by the ethics committees of all participating institutions (16). The study design is illustrated in Figure 1.

### Definitions and clinical endpoints

According to contemporary guidelines, AMI is defined as evidence of increased and decreased cardiac enzymes (i.e., troponin I) with at least one of the following scenarios: clinical manifestations indicative of myocardial ischemia; 12-lead electrocardiogram (ECG) results suggestive of myocardial ischemia, such as deviation of the ST-segment (elevation or decrease of the ST-segment) and/or development of pathological Q-waves; and characteristic features observed during cardiovascular imaging indicative of AMI (such as new loss of myocardial viability or newly detected regional wall motion abnormality). ST-segment elevation myocardial infarction (STEMI) refers to AMI with newly observed ST-segment elevation >1 mm (0.1 mV) in two or more contiguous leads, or newly observed left bundle branch block, on the surface of the 12-lead ECG.

Patients' clinical data, including demographic data, medical history, laboratory information, prescribed medications, and angiographic/echocardiographic information, were collected. According to a substudy of the HORIZON-AMI trial (17), baseline thrombocytopenia refers to platelet count <150 x 10^3^/μL in the blood sample obtained at the time of hospital presentation. The body mass index (kg/m^2^) was estimated using each patient's weight and height. A diagnosis of left main coronary artery (LMCA) disease necessitated angiographic confirmation of a reduction of ≥50% in the intraluminal diameter of the LMCA. Multivessel CAD was defined as an angiographic confirmation of ≥70% stenosis in two or more epicardial coronary arteries or ≥70% stenosis in one epicardial coronary artery with ≥50% stenosis of the LMCA. An infarct-related artery is an epicardial coronary vessel blocked or stenosed by an atheromatous or thrombotic pathologic process considered directly responsible for myocardial ischemia. The degree of coronary flow was categorized according to the thrombolysis in myocardial infarction (TIMI) flow grade (TIMI 0–I vs. TIMI II–III). Image-guided PCI was defined as any utilization of intracoronary imaging, such as optical coherence tomography or intravascular ultrasound, during PCI. Thrombus aspiration refers to a catheter-based manual aspiration procedure to improve myocardial perfusion during PCI. The left ventricular ejection fraction (LVEF) was estimated using two-dimensional echocardiography.

All survivors were clinically followed up for 36 months after discharge. The primary outcome was the occurrence of major adverse cardiac and cerebrovascular events (MACCEs). MACCEs comprise all-cause death, non-fatal myocardial infarction, any need for revascularization, and cerebrovascular accident (CVA). All-cause death comprises cardiac and non-cardiac deaths. Further, any need for revascularization was defined as any occurrence of repeat PCI or coronary artery bypass graft. CVA was defined as any cerebrovascular event, including hemorrhagic/ischemic stroke and transient ischemic attack.

### Ethics approval

The present study was conducted according to the ethical principles of the Declaration of Helsinki. This study was based on the KAMIR-NIH registry, and the study protocol for KAMIR-NIH was previously published and approved by the ethics committee of each participating medical center. The present study was approved by the Ethics Committee of Chonnam National University Hospital (CNUH-2021–360).

### Statistical analysis

Statistical analyses were conducted to evaluate the differences in clinical outcomes between the groups (group A and group B). Continuous variables are presented as means ± standard deviation, and discrete (categorical) variables are presented as numbers and percentages. Continuous variables were analyzed with Student's *t*-test, and discrete variables were analyzed with Pearson's chi-square test or Fisher's two-by-two exact test. All results were considered statistically significant when *p* < 0.05.

To minimize the effect of selection bias on the statistical analysis of the observational registry, we used two propensity score weighting methods (i.e., propensity score matching [PSM] and inverse probability of treatment weighting [IPTW]). The propensity score was constructed with a multiple logistic regression model using the following covariates: sex; age 75 years or older; Killip functional class III–IV (Killip I–II vs. Killip III–IV); body mass index ≥25 kg/m^2^; medical history (hypertension, diabetes mellitus, dyslipidemia, previous myocardial infarction, and previous CVA); smoking history (current smoker or former smoker vs. non-smoker); family history of CAD; laboratory profiles (white blood cell count, neutrophil-to-lymphocyte ratio [NLR] ≥2.5, hemoglobin count, platelet count, serum glucose, and serum creatinine); discharge medications (aspirin, calcium channel blockers, beta-blockers, angiotensin-converting enzyme inhibitors/angiotensin receptor blockers, and statins); angiographic profiles, including the vascular approach (femoral route vs. non-femoral route); use of glycoprotein IIb/IIIa inhibitors; use of thrombus aspiration; use of image-guided PCI; infarct-related artery; American College of Cardiology/American Heart Association (ACC/AHA) lesion types (ACC/AHA type B2/C vs. ACC/AHA type A/B1); TIMI flow grade (TIMI 0–I versus TIMI II–III); use of thrombolysis; LMCA disease; multivessel CAD; LVEF <40%; and final diagnosis (STEMI vs. non-STEMI). Patients with missing covariate data were excluded from the PSM- and IPTW-adjusted statistical analyses.

Cumulative events were analyzed using time-to-event data and the Kaplan–Meier method. All subjects were censored at the time of the event or the final follow-up. The survival curves were plotted according to the time of clinical outcomes and statistically compared using the log-rank test. All analyses were performed using SPSS version 25.0 (IBM SPSS Inc., Armonk, NY, United States).

## Results

### Baseline characteristics

The overall analysis included 800 consecutive Korean AMI patients with coexisting baseline thrombocytopenia. The baseline characteristics of these patients are shown in Table 1.

**Table 1:** Baseline characteristics of the study population.

	**Before propensity score weighting**			**After PSM**			**After IPTW**		
**Characteristics**	**Group A**	**Group B**	***p*-value**	**Group A**	**Group B**	***p*-value**	**Group A**	**Group B**	***p*-value**
	**(*n* = 244)**	**(*n* = 556)**		**(*n* = 174)**	**(*n* = 174)**		**(*n* = 630)**	**(*n* = 659)**	
Male patients	210 (86.1%)	432 (77.7%)	**0.006**	148 (85.1)	147 (84.5)	0.881	507 (80.5)	530 (80.4)	0.988
Age, years	64.58 ± 11.61	70.94 ± 11.37	**<0.001**	65.18 ± 12.08	65.20 ± 11.30	0.989	68.16 ± 12.42	68.95 ± 11.75	0.535
Age ≥ 75 years	48 (19.7%)	233 (41.9%)	**<0.001**	38 (21.8)	30 (17.2)	0.279	216 (34.3)	226 (34.3)	0.998
Killip classification			**0.018**			0.752			0.79
Killip class I-II	210 (86.1%)	439 (79.0%)		150 (86.2)	152 (87.4)		532 (84.5)	550 (83.5)	
Killip class III-IV	34 (13.9%)	117 (21.0%)		24 (13.8)	22 (12.6)		98 (15.5)	109 (16.5)	
BMI ≥ 25 kg/m^2^	88 (36.7%)	147 (27.9%)	**0.015**	65 (37.4)	62 (35.6)	0.738	197 (31.3)	206 (31.3)	0.998
**Previous medical history**									
Hypertension	117 (48.0%)	320 (57.6%)	**0.012**	87 (50.0)	81 (46.5)	0.520	356 (56.5)	355 (53.9)	0.598
Diabetes mellitus	91 (37.3%)	203 (36.5%)	0.832	69 (39.7)	66 (37.9)	0.741	254 (40.3)	256 (38.9)	0.768
Dyslipidemia	16 (6.6%)	59 (10.6%)	0.07	12 (6.9)	9 (5.2)	0.499	37 (5.8)	59 (9.0)	0.177
Prior MI	22 (9.0%)	55 (9.9%)	0.699	16 (9.2)	16 (9.2)	1.000	61 (9.7)	66 (10.0)	0.924
Old CVA	12 (5.0%)	71 (12.9%)	**0.001**	10 (5.7)	7 (4.0)	0.456	55 (8.7)	70 (10.7)	0.579
Smoking	147 (61.5%)	299 (54.9%)	0.084	99 (56.9)	105 (60.3)	0.514	329 (52.2)	379 (57.5)	0.283
Family history of CAD	19 (8.0%)	26 (4.8%)	0.077	11 (6.3)	13 (7.5)	0.672	50 (8.0)	37 (5.6)	0.38
**Laboratory profiles**									
WBC, 10^3^/μL	8.97 ± 3.56	9.44 ± 10.93	0.514	8.66 ± 3.35	8.64 ± 4.22	0.948	8.74 ± 3.50	9.22 ± 10.31	0.352
NLR ≥2.5	133 (54.5%)	364 (65.5%)	**0.003**	95 (54.6)	94 (54.0)	0.914	376 (59.6)	404 (61.3)	0.732
Hemoglobin, g/dL	13.74 ± 2.31	12.73 ± 2.44	**<0.001**	13.67 ± 2.37	13.70 ± 2.19	0.895	13.07 ± 2.59	13.12 ± 2.40	0.84
Platelet, 10^3^/μL	126.28 ± 24.19	120.89 ± 26.40	**0.007**	127.12 ± 20.69	126.25 ± 20.75	0.696	124.72 ± 23.09	123.34 ± 24.50	0.527
Glucose, mg/dL	180.55 ± 93.13	168.22 ± 83.80	0.069	179.94 ± 93.10	180.84 ± 101.06	0.931	179.88 ± 87.25	173.41 ± 91.94	0.452
Creatinine, mg/dL	1.31 ± 1.34	1.66 ± 1.89	**0.003**	1.38 ± 1.52	1.25 ± 1.46	0.439	1.55 ± 1.68	1.52 ± 1.80	0.88
**Discharge medications**									
Aspirin	243 (99.6%)	556 (100.0%)	0.305	174 (100.0)	174 (100.0)	1.000	630 (100.0)	659 (100.0)	1
Calcium channel blockers	7 (2.9%)	50 (9.0%)	**0.002**	7 (4.0)	10 (5.7)	0.456	54 (8.5)	51 (7.7)	0.818
Beta-blockers	198 (81.1%)	419 (75.4%)	0.073	145 (83.3)	142 (81.6)	0.672	498 (79.0)	529 (80.3)	0.75
ACEi or ARB	190 (77.9%)	408 (73.4%)	0.179	140 (80.5)	139 (79.9)	0.893	488 (77.4)	513 (77.9)	0.903
Statins	222 (91.0%)	271 (84.7%)	**0.016**	159 (91.4)	159 (91.4)	1.000	566 (89.8)	587 (89.0)	0.825
**Angiographic profiles**									
Transfemoral approach	136 (55.7%)	386 (69.4%)	**<0.001**	103 (59.2)	104 (59.8)	0.913	391 (62.0)	424 (64.3)	0.621
GPIIb/IIIa inhibitors	36 (14.8%)	67 (12.1%)	0.293	23 (13.2)	20 (11.5)	0.625	69 (10.9)	79 (11.9)	0.712
Thrombus aspiration	65 (26.6%)	120 (21.6%)	0.118	41 (23.6)	38 (21.8)	0.701	131 (20.8)	142 (21.5)	0.846
Image-guided PCI	61 (25.0%)	122 (21.9%)	0.343	38 (21.8)	48 (27.6)	0.214	148 (23.5)	151 (23.0)	0.897
Use of thrombolysis	0 (0.0%)	6 (1.1%)	0.103	0 (0.0)	0 (0.0)	1.000	0 (0.0)	0 (0.0)	1
ACC/AHA lesion type B2/C	112 (45.9%)	206 (37.1%)	**0.019**	83 (47.7)	74 (42.5)	0.332	268 (42.5)	272 (41.3)	0.804
Infarct-related artery			0.161			0.135			0.214
LMCA	7 (2.9%)	28 (5.0%)		5 (2.9)	8 (4.6)		13 (2.1)	24 (3.6)	
LAD	110 (45.1%)	215 (38.7%)		78 (44.8)	69 (39.7)		284 (45.1)	254 (38.5)	
LCX	33 (13.5%)	97 (17.4%)		23 (13.2)	38 (21.8)		79 (12.6)	120 (18.2)	
RCA	94 (38.5%)	216 (38.8%)		68 (39.1)	59 (33.9)		254 (40.2)	261 (39.7)	
TIMI flow grade 0-I	120 (49.2%)	286 (51.4%)	0.556	91 (52.3)	82 (47.1)	0.335	323 (51.2)	328 (49.8)	0.78
LMCA disease	13 (5.3%)	49 (8.8%)	0.09	9 (5.2)	13 (7.5)	0.378	31 (5.0)	42 (6.4)	0.499
Multivessel CAD	128 (52.5%)	314 (56.5%)	0.293	92 (52.9)	98 (56.3)	0.518	351 (55.7)	364 (55.2)	0.932
LVEF <40%	31 (13.4%)	108 (21.3%)	**0.011**	25 (14.4)	24 (13.8)	0.878	128 (20.4)	120 (18.3)	0.638
STEMI diagnosis	124 (50.8%)	237 (42.6%)	**0.032**	84 (48.3)	76 (43.7)	0.390	276 (43.7)	277 (42.0)	0.722

Values are presented as number (percentage) for categorical values and means ± standard deviation for continuous variables.

ACC/AHA, American College of Cardiology/American Heart Association; ACEi, angiotensin-converting enzyme inhibitor; ARB, angiotensin receptor blocker; BMI, body-mass index; CAD, coronary artery disease; CVA, cerebrovascular accidents; GPIIb/IIIa, glycoprotein IIb/IIIa; IPTW, inverse probability of treatment weighting; LAD, left anterior descending coronary artery; LCX, left circumflex coronary artery; LMCA, left main coronary artery; LVEF, left ventricular ejection fraction; MI, myocardial infarction; NLR, neutrophil-to-lymphocyte ratio; PCI, percutaneous coronary intervention; PSM, propensity score matching; RCA, right coronary artery; STEMI, ST-segment elevation myocardial infarction; TIMI, Thrombolysis In Myocardial Infarction; WBC, white blood cell.

The bold values indicates the statistical significance at the value of *p* <0.05.

Group A had more males (*p* = 0.006), obese patients (body mass index ≥25 kg/m^2^; *p* = 0.015), fewer patients aged 75 years or older (*p* < 0.001) and patients with Killip functional class III–IV (*p* = 0.018) than group B. Hypertension (*p* = 0.012) and previous CVA (*p* = 0.001) were more prevalent in group B than in group A. In terms of laboratory profiles, group A had fewer patients with NLR ≥2.5 (*p* = 0.003) and higher creatinine (*p* = 0.003), hemoglobin (*p* < 0.001), and platelet (*p* = 0.007) levels than group B. Regarding discharge medications, group A patients were less likely to be prescribed calcium channel blockers (*p* = 0.002) and more likely to be prescribed statins (*p* = 0.016) than group B patients. Regarding angiographic profiles, the transfemoral approach was used more frequently for group B than for group A (*p* < 0.001). Group A had more patients with ACC/AHA type B2/C than group B (*p* = 0.019). Additionally, group A had fewer patients with LVEF <40% than group B (*p* = 0.011). STEMI was more prevalent in group A than in group B (*p* = 0.032). To overcome the baseline differences between two groups, both PSM and IPTW was introduced. After PSM and IPTW adjustment, all the aforementioned differences were adequately balanced between the two groups (the distribution of the absolute standardized differences for each covariate is illustrated in Supplementary Figure 1 and Supplementary Table 1).

### Long-term clinical outcomes

**Figure 2:**
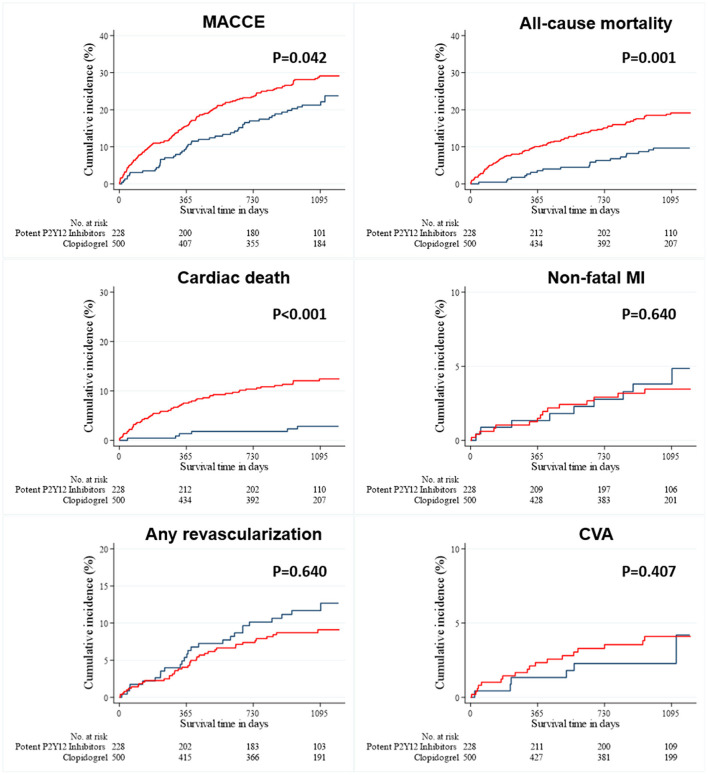
Event rates of clinical outcomes for all the patients after a 3-year follow-up (before PSM-and IPTW-adjusted analysis). The Kaplan–Meier curves for cumulative event rates according to the type of P2Y12 receptor inhibitors are shown. The red curve indicates group A, and the blue curve indicates group B. CVA, cerebrovascular accident; IPTW, inverse probability of treatment weighting; MACCE, major adverse cardiac and cerebrovascular event; MI, myocardial infarction.

**Figure 3:**
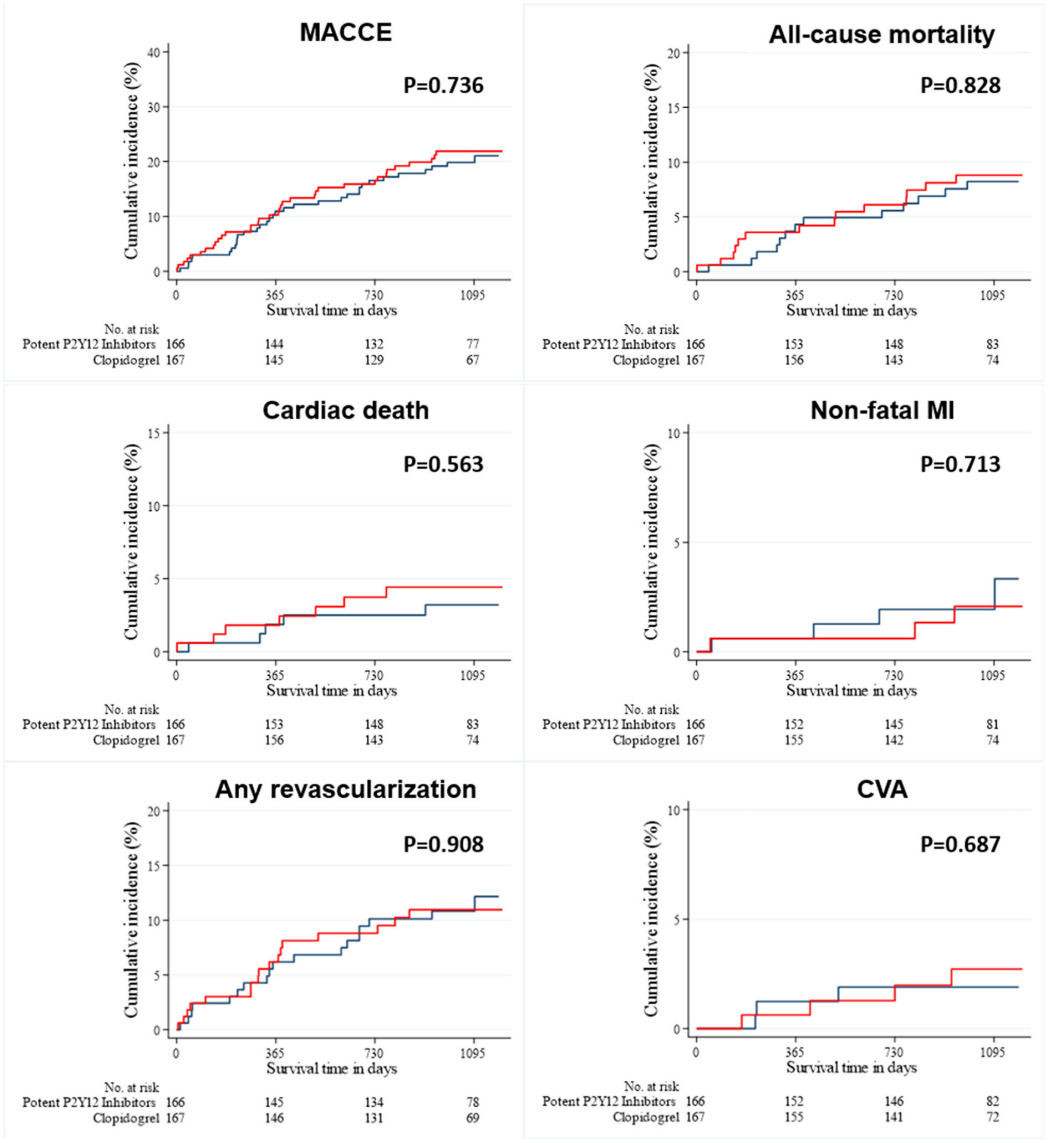
Event rates of clinical outcomes for all patients after a 3-year follow-up (after PSM-adjusted analysis). The Kaplan–Meier curves for cumulative event rates according to the type of P2Y12 receptor inhibitors are shown. The red curve indicates group A, and the blue curve indicates group B. CVA, cerebrovascular accident; MACCE, major adverse cardiac and cerebrovascular event; MI, myocardial infarction; PSM, propensity score matching.

**Figure 4:**
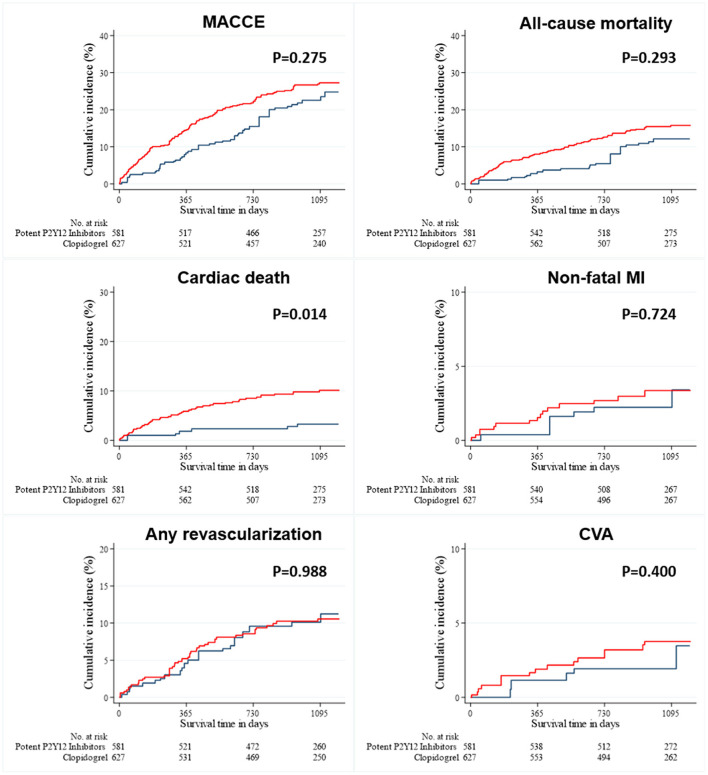
Event rates of clinical outcomes for all patients after a 3-year follow-up (after IPTW-adjusted analysis). The Kaplan–Meier curves for cumulative event rates according to the type of P2Y12 receptor inhibitors are shown. The red curve indicates group A, and the blue curve indicates group B. CVA, cerebrovascular accident; IPTW, inverse probability of treatment weighting; MACCE, major adverse cardiac and cerebrovascular event; MI, myocardial infarction.

As shown in Figure 1, long-term clinical outcomes of subjects who were successfully discharged and followed-up as outpatients were analyzed. The median follow-up interval was 1,085 days. Clinical outcomes that occurred during the 36-months follow-up interval are summarized in Table 2; these include MACCEs, all-cause death, cardiac death, non-fatal MI, the need for revascularization, and CVA. The survival curves were plotted to illustrate the unadjusted (Figure 2), PSM-adjusted (Figure 3), and IPTW-adjusted analyses (Figure 4). After PSM and IPTW adjustment, no significant differences were observed between groups; however, the IPTW-adjusted analysis showed that the incidence of cardiac death was lower in group A than in group B.

**Table 2:** Three-year clinical outcomes in propensity score matched patients.

**Outcomes**	**Group A**	**Group B**	**Unadjusted analysis**		**PSM-adjusted analysis**		**IPTW-adjusted analysis**	
	**(*n* = 228)**	**(*n* = 500)**	**HR (95% CI) (a)**	***p*-value**	**HR (95% CI) (b)**	***p*-value**	**HR (95% CI) (b)**	***p*-value**
MACCE (c)	49/228 (21.5%)	139/500 (27.8%)	1.399 (1.010–1.938)	**0.043**	1.085 (0.674–1.746)	0.736	1.252 (0.836–1.875)	0.275
All-cause death	21/228 (9.2%)	91/500 (18.2%)	2.138 (1.330–3.437)	**0.002**	1.087 (0.511–2.313)	0.828	1.397 (0.749–2.607)	0.293
Cardiac death	6/228 (2.6%)	58/500 (11.6%)	4.716 (2.035–10.930)	**<0.001**	1.403 (0.445–4.421)	0.563	3.218 (1.268–8.171)	**0.014**
Non-cardiac death	15/228 (6.6%)	33/500 (6.6%)	1.102 (0.599–2.029)	0.755	0.888 (0.322–2.450)	0.819	0.718 (0.314–1.644)	0.433
Non-fatal MI	9/228 (3.9%)	15/500 (3.0%)	0.821 (0.359–1.877)	0.641	0.756 (0.169–3.376)	0.714	1.233 (0.385-3.952)	0.724
Any revascularization	26/228 (11.4%)	39/500 (7.8%)	0.735 (0.448–1.208)	0.225	0.962 (0.496–1.866)	0.908	0.995 (0.556–1.782)	0.988
Re-do PCI	24/228 (10.5%)	37/500 (7.4%)	0.760 (0.455–1.270)	0.295	0.901 (0.450–1.804)	0.768	1.002 (0.550–1.824)	0.996
CABG	3/228 (1.3%)	2/500 (0.4%)	0.338 (0.056–2.021)	0.234	2.061 (0.187–22.731)	0.555	0.959 (0.087–10.618)	0.973
CVA	6/228 (2.6%)	18/500 (3.6%)	1.475 (0.585–3.716)	0.410	1.361 (0.305–6.080)	0.687	1.602 (0.534–4.802)	0.400

Values are presented as percentage (number) for categorical values.

CABG, coronary artery bypass graft; CAD, coronary artery disease; CI, confidence interval; CVA, cerebrovascular accident; HR, hazard ratio; IPTW, inverse probability of treatment weighting; MACCE, major adverse cardiac and cerebrovascular event; MI, myocardial infarction; PCI, percutaneous coronary intervention; PSM, propensity score matching; STEMI, ST-segment elevation myocardial infarction.

(a) HR corresponds to group B compared with group A. (b) Adjusted Cox hazard regression analysis included a variety of clinical variables, including age, Killip functional class, body-mass index, hypertension, diabetes mellitus, dyslipidemia, prior MI, old CVA, smoking history, family CAD history, white blood cell, neutrophil-to-lymphocyte ratio, hemoglobin, platelet, glucose, creatinine, group (group A vs. group B), aspirin, calcium channel blockers, beta-blockers, angiotensin-converting enzyme inhibitors/angiotensin receptor blockers, statins, transfemoral approach, glycoprotein IIb/IIIa inhibitors, thrombus aspiration, image-guided PCI, infarct-related artery, American College of Cardiology/American Heart Association lesion types, Thrombolysis In Myocardial Infarction flow grade 0-I, thrombolysis, left main coronary artery disease, multivessel CAD, left ventricular ejection fraction, STEMI diagnosis. (c) MACCE is defined as a composite of all-cause death, non-fatal myocardial infarction, any revascularization, and CVA.

The bold values indicates the statistical significance at the value of *p* < 0.05.

## Discussion

We compared the 3-years clinical outcomes of Korean AMI patients with baseline thrombocytopenia administered potent P2Y12 inhibitors (group A) and those of Korean AMI patients with baseline thrombocytopenia administered clopidogrel (group B). In the unadjusted analysis, group A had fewer MACCEs and lower all-cause death, and cardiac death rates than group B during the 3-years follow-up period. Although all the differences were statistically attenuated after the PSM-adjusted analysis, only the cardiac death rate remained lower in group A than in group B after the IPTW-adjusted analysis.

We found that clopidogrel was administered more than twice as often as potent P2Y12 inhibitors (ticagrelor and prasugrel) to the overall population. This finding reflects the real-world clinical practice of considering the bleeding tendencies of these selected AMI patients. Group A included more males more younger individuals and more obese individuals than group B. This may be related to the fact that prasugrel is not generally recommended for older adults (age 75 years or older) or patients with lower body weight (18). Hypertension and previous CVA were less prevalent in group A than in group B. When interpreting this finding, the CVA-related contraindications of these potent P2Y12 inhibitors should be considered. In fact, ticagrelor is contraindicated for patients with a history of intracranial hemorrhage, and prasugrel is contraindicated for patients with a history of stroke or transient ischemic attack (1, 10). Group A had lower levels of NLR and creatinine but higher levels of hemoglobin and platelets than group B. This indicates that ticagrelor or prasugrel was administered to patients with less inflammation (lower NLR levels), a better kidney function (lower creatinine levels), and lower bleeding tendency (higher hemoglobin and platelet levels). Group A was administered calcium channel blockers at a lower frequency than group B at discharge. However, group A was administered statins at a higher frequency than group B. Group A tended to have more complex coronary lesions and a higher incidence of ACC/AHA lesion type B2/C than group B. In contrast, fewer transfemoral approaches were chosen for group A than for group B. Group A had relatively higher LVEF values than group B. However, group A had a higher proportion of STEMI patients than group B.

The 2017 European Society of Cardiology STEMI guidelines and 2020 European Society of Cardiology non-ST-segment elevation acute coronary syndrome guidelines recommend the clinical application of a potent P2Y12 inhibitor (i.e., ticagrelor or prasugrel) for AMI patients who have undergone PCI (1, 10). Compared to clopidogrel, these potent P2Y12 inhibitors are beneficial in CAD, reducing its mortality rate (19). Despite these recommendations and benefits, many clinicians may be reluctant to prescribe these drugs to patients with low platelet levels at the time of admission because of concerns regarding bleeding tendencies. Another critical point to consider involves ethnic differences. East Asian patients tend to experience fewer ischemic events and more bleeding events after PCI (3). Park et al. evaluated an East Asian population with acute coronary syndrome and found that ticagrelor was associated with clinically significant bleeding compared to clopidogrel (10). Considering antiplatelet agent response differences between East Asians and Caucasians, many clinicians selected clopidogrel instead of potent P2Y12 inhibitors for AMI patients involved in this study. Although the unadjusted analyses performed in this study showed that using a potent P2Y12 inhibitor—in addition to aspirin—may seemingly improve outcomes in these patients, both PSM-adjusted and IPTW-adjusted analyses suggest that potent P2Y12 inhibitors have no obvious benefits over clopidogrel.

In addition to the 3-year clinical outcomes, we examined the in-hospital clinical outcomes of both groups (Supplementary Table 2). Group A and group B had similar rates of in-hospital complications, particularly bleeding complications. This suggests that both ticagrelor and prasugrel are relatively safer than expected, at least in terms of short-term bleeding complications. Interestingly, this is contrary to the results of previous studies that found that both drugs were associated with high bleeding tendencies for Korean AMI patients (20, 21). Nevertheless, because comparative analysis of long-term bleeding outcomes was not conducted during this study, it is difficult to conclude that the two antiplatelet agents have similar safety outcomes based on the results.

We further investigated the distribution according to platelet counts for all subjects enrolled in this study. As presented in Supplementary Table 3, 85.0% of the overall study patients had platelet levels ≥100 x 10^3^/μL and <150 x 10^3^/μL. In contrast, only 3.1% (25) of 800 patients with platelet levels <50 x 10^3^/μL (i.e., severe thrombocytopenia) were present. Because the total number of patients in the KAMIR-NIH registry is 13,104, this was an extremely small proportion of the AMI population. However, because these patients are indeed at a greater risk of bleeding diathesis and represent a very small percentage of the overall study population, we must be careful when interpreting the magnitude of results of the present study. Further studies of AMI patients with severe thrombocytopenia are needed in the future. In these subgroups, most MACCEs were comparable between group A and group B, except for the IPTW-adjusted analysis of the patients with platelet levels ≥50 x 10^3^/μL and <100 x 10^3^/μL. This sensitivity analysis is shown in Supplementary Table 4. Among these patients, different incidences of MACCEs were related to different incidences of all-cause death and cardiac death. However, the PSM-adjusted analysis of the same population showed no such difference. To elucidate these heterogenous results, further investigation is needed.

According to the TRITON-TIMI and PLATO trials, potent P2Y12 inhibitors are better for reducing the incidence of adverse ischemic events than clopidogrel (22, 23). Nonetheless, according to two clinical studies based on the KAMIR-NIH registry, the beneficial effects of potent P2Y12 inhibitors compared to those of clopidogrel appeared attenuated among Korean AMI patients (20, 21). The lower risk of atherothrombotic events and higher risk of bleeding complications among East Asian patients compared to those of Caucasians constitute the East Asian paradox (3). Awareness of this supposed paradox may reinforce the belief that clopidogrel is a better option than ticagrelor or prasugrel for East Asian AMI patients with thrombocytopenia who are presumably at higher risk for bleeding. We observed that the administration of clopidogrel was associated with clinical outcomes relatively comparable to anti-ischemic events similar to those of potent P2Y12 inhibitors. Because these two antiplatelet agents resulted in similar ischemic outcomes, as presented by a study based on the KAMIR-NIH registry (3), our results seem consistent with the idea of the East Asian paradox. In other words, the principle of the similar ischemic potency between potent P2Y12 inhibitors and clopidogrel is robustly maintained for Korean AMI patients with baseline thrombocytopenia. Therefore, it seems reasonable to use clopidogrel instead of ticagrelor or prasugrel for this selected group of patients.

Because the present study demonstrates that clopidogrel is a good and reasonable therapeutic option with MACCE rates similar to those of potent P2Y12 inhibitors for Korean AMI patients with baseline thrombocytopenia, it fully supports the concept of the East Asian paradox, even in this clinical setting. However, our results should be interpreted with caution for the following reasons. First, the KAMIR-NIH registry only included the data of AMI patients from tertiary cardiovascular medical centers that frequently perform PCI procedures. Consequently, it is difficult to generalize these clinical results, including adverse cardiovascular events and treatment patterns, to all medical centers treating AMI patients. Additionally, the results of the present study were based on observational data derived from the Korean population, but not from other Asian populations, such as Japanese or Chinese. Although our results may be clinically important, additional information on other Asian populations other than Koreans is necessary. Second, several variables were missing in the present study that may influence clinical results and could be considered clinically important. We did not collect detailed information about antiplatelet agents, such as DAPT duration or transitions to another antiplatelet agent. Unfortunately, we did not collect data regarding bleeding events during follow-up. Therefore, since both thrombotic and hemorrhagic risk was not considered, we did not demonstrate the long-term safety outcomes of both types of antiplatelet agents. Moreover, no data on the prevalence of atrial fibrillation was available in the present study, one of the crucial factors influencing the selection of antithrombotic agents. Third, as previously mentioned, this study included patients with relatively mild thrombocytopenia. Since only 3.1% of the study population had a platelet level <50 x 10^3^/μL, it remains unclear whether an even better relationship exists between the types of P2Y12 inhibitors and clinical outcomes of patients with AMI and severe thrombocytopenia. In addition, considering that various medical conditions or medications can cause thrombocytopenia, this study population can be considered different or heterogeneous based on the underlying causes of baseline thrombocytopenia. Therefore, an individualized and multidisciplinary approach is first required in actual clinical practice before implementing the study results. Fourth, although this study was based on the KAMIR-NIH registry, a nationwide, multicenter, observational registry, it was not randomized, thus producing selection bias in the statistical analysis. Although two propensity score weighting methods, PSM and IPTW, were used to adjust for potential selection bias when the treatment outcome was dependent on the measured patient's baseline characteristics, the problem of selection bias may have remained because of the inclusion and exclusion criteria, exclusion of data with missing values, and many unmeasured confounders. Therefore, our results may not present the causal relationship between the P2Y12 inhibitor types and treatment estimates. Hence, a multicenter, randomized controlled trial is necessary to compare clinical outcomes of the two types of antiplatelet agents.

## Conclusions

Until now, the optimal antiplatelet therapy for AMI and baseline thrombocytopenia has been inadequately studied and poorly understood. Using the KAMIR-NIH registry, a large registry of Korean AMI patient data, we found that the use of clopidogrel was reasonable and associated with outcomes comparable to those of potent P2Y12 inhibitors for patients with low platelet levels at presentation. Our results will influence future guidelines for treating East Asian (specifically Korean) AMI patients.

## Data availability statement

The raw data supporting the conclusions of this article will be made available by the authors, without undue reservation.

## Ethics statement

The studies involving human participants were reviewed and approved by the Institutional Review Board of Chonnam National University Hospital. Written informed consent for participation was not required for this study in accordance with the national legislation and the institutional requirements.

## Author contributions

Conceptualization, methodology, investigation, data curation, writing—original draft preparation, and project administration: SO. Resources and writing—review and editing: SO, KC, MK, DS, YH, JK, YA, and MJ. All authors have read and agreed to the published version of the manuscript.

## Funding

This study was supported by grants from the Korean Health Technology R&D Project, Ministry of Health and Welfare (HI13C1527) and the Research of Korea Centers for Disease Control and Prevention (2016-ER6304-01), Republic of Korea.

## Conflict of interest

The authors declare that the research was conducted in the absence of any commercial or financial relationships that could be construed as a potential conflict of interest.

## Publisher's note

All claims expressed in this article are solely those of the authors and do not necessarily represent those of their affiliated organizations, or those of the publisher, the editors and the reviewers. Any product that may be evaluated in this article, or claim that may be made by its manufacturer, is not guaranteed or endorsed by the publisher.
