# Supplementary material for: Impact of P2Y12 inhibitors on cardiovascular outcomes of Korean acute myocardial infarction patients with baseline thrombocytopenia
Source: Front Cardiovasc Med. 2022 Sep 14;9:921955. doi: 10.3389/fcvm.2022.921955 (PMC9515375; doi:10.3389/fcvm.2022.921955)
Supplement: Supplementary file 1 [file Table_1.docx]

**Supplementary Table 1**. Distribution of absolute standardized differences

|  | Standardized differences | | |
| --- | --- | --- | --- |
|  | **Unadjusted data** | **PSM-adjusted data** | **IPTW-adjusted data** |
| Male patients | 18.815 | 15.404 | 8.584 |
| Age ≥ 75 years | 53.131 | 14.656 | 1.939 |
| Killip class III-IV | 11.901 | 6.876 | 3.681 |
| BMI ≥25 kg/m2 | 18.356 | 1.665 | 1.676 |
| Hypertension | 15.474 | 15.179 | 6.304 |
| Diabetes mellitus | 3.032 | 5.995 | 0.343 |
| Dyslipidemia | 15.999 | 20.107 | 12.010 |
| Prior MI | 0.699 | 5.714 | 0.236 |
| Old CVA | 30.782 | 4.325 | 3.410 |
| Smoking | 8.382 | 16.927 | 12.296 |
| Family history of CAD | 17.396 | 1.289 | 9.175 |
| WBC | 4.560 | 7.306 | 6.785 |
| NLR ≥2.5 | 30.353 | 3.103 | 4.627 |
| Hemoglobin | 40.156 | 10.975 | 3.765 |
| Platelet | 21.148 | 0.389 | 7.285 |
| Glucose | 19.493 | 4.937 | 5.232 |
| Creatinine | 16.896 | 0.881 | 0.927 |
| Calcium channel blockers | 26.005 | 11.788 | 1.198 |
| Beta-blockers | 5.739 | 1.526 | 0.134 |
| ACEi or ARB | 4.582 | 5.853 | 1.832 |
| Statins | 14.308 | 6.754 | 3.503 |
| Transfemoral approach | 22.649 | 1.898 | 4.274 |
| GPIIb/IIIa inhibitors | 7.453 | 7.671 | 2.221 |
| Thrombus aspiration | 7.707 | 2.891 | 2.799 |
| Image-guided PCI | 3.919 | 7.873 | 0.047 |
| LAD | 15.534 | 4.714 | 4.761 |
| LCX | 12.490 | 3.155 | 2.445 |
| RCA | 3.882 | 3.724 | 4.549 |
| ACC/AHA lesion type B2/C | 23.209 | 24.717 | 4.086 |
| TIMI flow grade 0-I | 4.212 | 3.052 | 3.431 |
| LMCA disease | 9.113 | 6.612 | 7.260 |
| Multivessel CAD | 7.875 | 12.274 | 1.170 |
| LVEF <40% | 18.522 | 3.097 | 3.260 |
| STEMI diagnosis | 20.357 | 1.555 | 3.300 |

All values are presented as percentage.

ACC/AHA = American College of Cardiology/American Heart Association; ACEi = angiotensin-converting enzyme inhibitor; ARB = angiotensin receptor blocker; BMI = body-mass index; CAD = coronary artery disease; CVA = cerebrovascular accidents; GPIIb/IIIa = glycoprotein IIb/IIIa; LAD = left anterior descending coronary artery; LCX = left circumflex coronary artery; LMCA = left main coronary artery; LVEF = left ventricular ejection fraction; MI = myocardial infarction; NLR = neutrophil-to-lymphocyte ratio; PCI = percutaneous coronary intervention; RCA = right coronary artery; STEMI = ST-segment elevation myocardial infarction; TIMI = Thrombolysis In Myocardial Infarction; WBC = white blood cell.
