# Supplementary material for: Impact of P2Y12 inhibitors on cardiovascular outcomes of Korean acute myocardial infarction patients with baseline thrombocytopenia
Source: Front Cardiovasc Med. 2022 Sep 14;9:921955. doi: 10.3389/fcvm.2022.921955 (PMC9515375; doi:10.3389/fcvm.2022.921955)
Supplement: Supplementary file 2 [file Table_2.docx]

**Supplementary Table 2**. Short-term outcomes during index hospitalization, from 800 AMI patients with baseline thrombocytopenia

| Characteristics | Group A  (n = 244) | Group B  (n = 556) | *p*-value |
| --- | --- | --- | --- |
| In-hospital death | 14 (5.7%) | 48 (8.6%) | 0.159 |
| In-hospital complications |  |  |  |
| Cardiogenic shock | 32 (13.1%) | 89 (16.0%) | 0.293 |
| New-onset heart failure | 16 (6.6%) | 36 (6.5%) | 0.965 |
| Reoccur MI | 2 (0.8%) | 1 (0.2%) | 0.222 |
| Stent thrombosis | 2 (0.8%) | 1 (0.2%) | 0.222 |
| Any CVA | 5 (2.0%) | 18 (3.2%) | 0.354 |
| Bleeding complications  (Hgb decrease by 5 g/dL or Hct decrease by 15 %) | 5 (2.0%) | 15 (2.7%) | 0.588 |
| Ventricular tachycardia or fibrillation | 17 (7.0%) | 34 (6.1%) | 0.650 |
| New-onset atrial fibrillation | 19 (7.8%) | 27 (4.9%) | 0.101 |
| Acute kidney injury | 3 (1.2%) | 14 (2.5%) | 0.298 |
| Sepsis | 1 (0.4%) | 11 (2.0%) | 0.119 |
| Multi-organ failure | 3 (1.2%) | 19 (3.4%) | 0.101 |

Values are presented as number (percentage) for categorical values.

AMI = acute myocardial infarction; CVA = cerebrovascular accident; Hct = hematocrit; Hgb = hemoglobin; MI = myocardial infarction.
