# Supplementary material for: Impact of P2Y12 inhibitors on cardiovascular outcomes of Korean acute myocardial infarction patients with baseline thrombocytopenia
Source: Front Cardiovasc Med. 2022 Sep 14;9:921955. doi: 10.3389/fcvm.2022.921955 (PMC9515375; doi:10.3389/fcvm.2022.921955)
Supplement: Supplementary file 3 [file Table_3.docx]

**Supplementary Table 3**. The distribution of platelet counts, from 800 AMI patients with baseline thrombocytopenia

| Characteristics | Overall  (n = 800) | Group A  (n = 244) | Group B  (n = 556) | *p*-value |
| --- | --- | --- | --- | --- |
| Platelet level |  |  |  | 0.169 |
| Platelet <50 x 10^3^/μL | 25 (3.1%) | 7 (2.9%) | 18 (3.2%) |  |
| Platelet ≥50 x 10^3^/μL, and <100 x 10^3^/μL | 95 (11.9%) | 22 (9.0%) | 73 (13.1%) |  |
| Platelet ≥100 x 10^3^/μL, and <150 x 10^3^/μL | 680 (85.0%) | 215 (88.1%) | 465 (83.6%) |  |

Values are presented as number (percentage) for categorical values. AMI = acute myocardial infarction.
