# Supplementary material for: Impact of P2Y12 inhibitors on cardiovascular outcomes of Korean acute myocardial infarction patients with baseline thrombocytopenia
Source: Front Cardiovasc Med. 2022 Sep 14;9:921955. doi: 10.3389/fcvm.2022.921955 (PMC9515375; doi:10.3389/fcvm.2022.921955)
Supplement: Supplementary file 4 [file Table_4.docx]

**Supplemental Table 4**. Three-year clinical outcomes in three subgroups stratified by platelet counts.

| Outcomes | Group A | Group B | Unadjusted analysis | | PSM-adjusted analysis | | IPTW-adjusted analysis | |
| --- | --- | --- | --- | --- | --- | --- | --- | --- |
|  | **n=228** | **n=500** | HR (95% CI) ^a)^ | *p*-value | HR (95% CI) ^b)^ | *p*-value | HR (95% CI) ^b)^ | *p*-value |
| Platelet <50 x 10^3^/μL | | | | | | | | |
| MACCE^c)^ | 1/5 (20.0) | 5/14 (35.7) | 2.136 (0.248-18.437) | 0.490 | - | - | 1.387 (0.143-13.433) | 0.778 |
| All-cause death | 1/5 (20.0) | 5/14 (35.7) | 2.136 (0.248-18.437) | 0.490 | - | - | 1.387 (0.143-13.433) | 0.778 |
| Cardiac death | 0/5 (0.0) | 2/14 (14.3) | - | - | - | - | - | - |
| Non-cardiac death | 1/5 (20.0) | 3/14 (21.4) | 1.363 (0.140-13.275) | 0.790 | - | - | 0.942 (0.084-10.533) | 0.962 |
| Platelet ≥50 x 10^3^/μL, and <100 x 10^3^/μL | | | | | | | | |
| MACCE^c)^ | 4/20 (20.0) | 24/63 (38.1) | 2.461 (0.853-7.103) | 0.096 | 1.591 (0.354-7.154) | 0.545 | 5.369 (1.357-21.254) | **0.017** |
| All-cause death | 3/20 (15.0) | 16/63 (25.4) | 2.073 (0.603-7.123) | 0.247 | 1.177 (0.165-8.398) | 0.871 | 6.649 (1.374-32.185) | **0.019** |
| Cardiac death | 0/20 (0.0) | 7/63 (11.1) | - | - | - | - | - | - |
| Non-cardiac death | 3/20 (15.0) | 9/63 (14.3) | 1.187 (0.321-4.390) | 0.798 | 0.571 (0.051-6.337) | 0.648 | 3.553 (0.676-18.678) | 0.134 |
| Platelet ≥100 x 10^3^/μL, and <150 x 10^3^/μL | | | | | | | | |
| MACCE^c)^ | 44/203 (21.7) | 110/423 (26.0) | 1.269 (0.895-1.800) | 0.181 | 1.062 (0.640-1.762) | 0.816 | 1.063 (0.695-1.625) | 0.780 |
| All-cause death | 17/203 (8.4) | 70/423 (16.5) | 2.100 (1.236-3.567) | **0.006** | 1.179 (0.509-2.728) | 0.701 | 1.163 (0.592-2.283) | 0.661 |
| Cardiac death | 6/203 (3.0) | 49/423 (11.6) | 4.137 (1.772-9.658) | **0.001** | 1.177 (0.359-3.856) | 0.788 | 2.634 (1.030-6.735) | **0.043** |
| Non-cardiac death | 11/203 (5.4) | 21/423 (5.0) | 0.984 (0.475-2.042) | 0.966 | 1.180 (0.360-3.867) | 0.784 | 0.542 (0.211-1.394) | 0.204 |

Values are presented as percentage (number) for categorical values.

CAD = coronary artery disease; CI = confidence interval; CVA = cerebrovascular accident; HR = hazard ratio; IPTW= inverse probability of treatment weighting; MACCE = major adverse cardiac and cerebrovascular event; MI = myocardial infarction; PCI = percutaneous coronary intervention.

^a)^HR corresponds to group B compared with group A; ^b)^Adjusted Cox hazard regression analysis included a variety of clinical variables, including age, Killip functional class, body-mass index, hypertension, diabetes mellitus, dyslipidemia, prior MI, old CVA, smoking history, family CAD history, white blood cell, neutrophil-to-lymphocyte ratio, hemoglobin, platelet, glucose, creatinine, group (group A versus group B), aspirin, calcium channel blockers, beta-blockers, angiotensin-converting enzyme inhibitors/angiotensin receptor blockers, statins, transfemoral approach, glycoprotein IIb/IIIa inhibitors, thrombus aspiration, image-guided PCI, infarct-related artery, American College of Cardiology/American Heart Association lesion types, Thrombolysis In Myocardial Infarction flow grade 0-I, thrombolysis, left main coronary artery disease, multivessel CAD, left ventricular ejection fraction, STEMI diagnosis. **^c)^**MACCE is defined as a composite of all-cause death, non-fatal myocardial infarction, any revascularization, and CVA.
