# Supplementary figures and images for: Impact of P2Y12 inhibitors on cardiovascular outcomes of Korean acute myocardial infarction patients with baseline thrombocytopenia
Source: Front Cardiovasc Med. 2022 Sep 14;9:921955. doi: 10.3389/fcvm.2022.921955 (PMC9515375; doi:10.3389/fcvm.2022.921955)

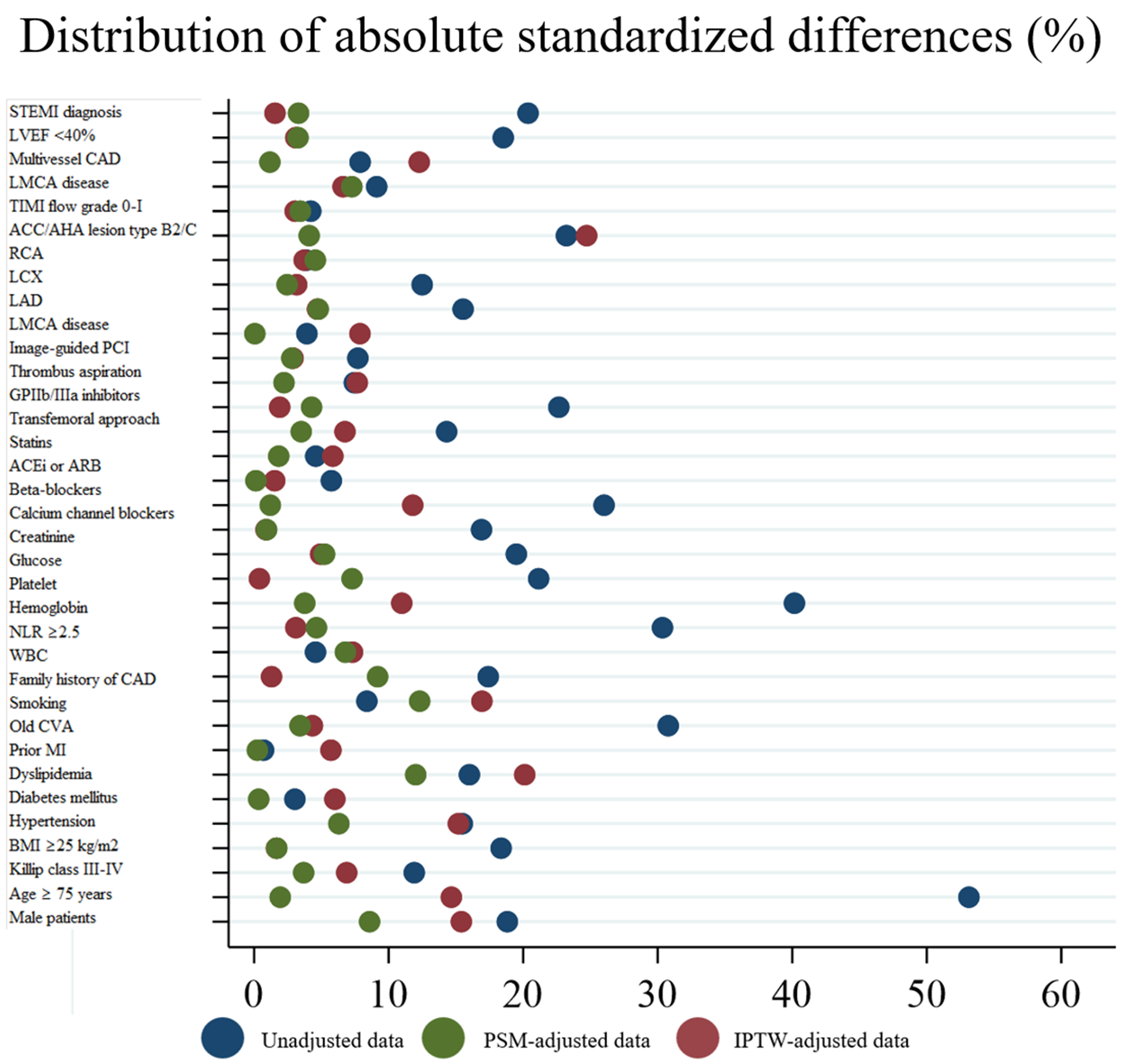

Supplement: Supplementary file 5 [file Image_1.TIF]
